# Exploiting Greener Formulations of Benzalkonium Chloride with Enhanced Antibacterial Properties

**DOI:** 10.1021/acsomega.5c10270

**Published:** 2026-03-11

**Authors:** Chiara Molinar, Giulia Vigna, Sara Scutera, Tiziana Musso, Anna Scomparin, Roberta Cavalli

**Affiliations:** † Department of Drug Science and Technology, 9314 University of Turin, Via P. Giuria 9, 10125 Turin, Italy; ‡ Department of Public Health and Pediatric Sciences, University of Turin, Via Santena 9, 10126 Turin, Italy

## Abstract

In recent years, the extensive use of benzalkonium chloride (BKC) as a disinfectant has raised significant concerns due to its toxicity and its potential to promote environmental impact and antimicrobial resistance. In order to reduce the toxicity associated with BKC in spray disinfectants, the antimicrobial agent was coencapsulated into vesicle-like nanosystems, known as nanodroplets (NDs), with limonene, a natural essential oil renowned for its antimicrobial properties and commonly used in cleaning product formulations. The aim was to reduce the required concentration of BKC in the disinfectant formulation by enhancing its antimicrobial activity through encapsulation in nanodroplets (BKC-NDs) and by loading limonene within the *core* of the nanodroplets. The vesicles consist of two distinct phases: an organic *core* composed of decafluoropentane and limonene and an outer aqueous shell stabilized at the interface by BKC. In this formulation, therefore, BKC plays both a functional role as an antimicrobial agent and a technological role as a surfactant, allowing for the formation of the NDs. Moreover, a more sustainable formulation was obtained by removing decafluoropentane and retaining limonene in the vesicle *core*. A library of BKC-NDs with varying BKC concentrations (0.008, 0.08, and 2.4% w/v), with or without a decafluoropentane *core*, was prepared to finally select the minimum concentration with the highest antimicrobial activity. BKC-NDs demonstrated superior antimicrobial properties compared to BKC in aqueous solution against *Staphylococcus aureus* and *Escherichia coli* and, in particular, the formulation at 0.08% w/v, while maintaining comparable *in vitro* biocompatibility. The efficiency of BKC-NDs spray disinfectants was also assessed from a physicochemical perspective, demonstrating high wettability, wide spray angle, and long-term stability upon storage while retaining antimicrobial efficacy. A novel green spray disinfectant based on BKC, which exploits materials of natural origin to reduce the concentration of chemicals, was developed to minimize its related toxicity and environmental contamination.

## Graphical abstract

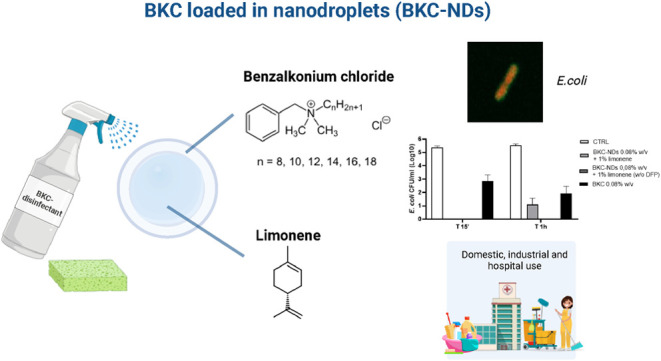

## 1 Introduction

Benzalkonium chloride (BKC) is one of the most widely used disinfectants, antiseptics, and preservatives that belong to the class of quaternary ammonium compounds (QACs).^1^ The distinctive chemical structure of QACs consists of a central quaternary ammonium cation attached to various alkyl chains that differ in type and in length.^2^ BKC is a mixture of alkylbenzyldimethylammonium chlorides with C12 to C18 alkyl chains. The unique amphiphilic nature of this class, combining a cationic head with a hydrophobic alkyl chain, gives them both antimicrobial activity and surfactant properties.^3^ Regarding its antimicrobial activity, BKC demonstrates broad-spectrum efficacy against Gram-positive and Gram-negative bacteria, fungi, parasites, and lipophilic (enveloped) viruses.^4^ The cationic head of BKC enables it to electrostatically bind to negatively charged microbial membranes and disrupt lipidic membranes.^5^ Acting as a cationic detergent, BKC is capable of lowering surface tension and of forming micelles, which further supports its disinfectant and antiseptic functions. Due to these properties, BKC is widely used in industrial, clinical, agricultural, and household products for cleaning and deodorizing purposes.^6^

Despite its widespread use in disinfectant and cleaning products, BKCs present several limitations, especially related to human toxicity and environmental concern.^7,8^ Indeed, BKC is classified as highly toxic to aquatic life (H400) with long-lasting effects (H410), as its residues can accumulate in sediments and water bodies from various human activities, adding significantly to environmental contamination.^9,10^ Compounding the problem, the environmental accumulation can promote the development of decreased susceptibility to both disinfectants and antibiotics.^4,11,12^

BKC and similar biocides have come under scrutiny due to potential health and environmental risks. In the United States, concerns over insufficient safety and efficacy data led the FDA in 2016 to prohibit 19 active ingredients in antiseptic consumer washes; BKC was not immediately banned but was deferred for further evaluation.^13^ In the European Union, BKC is still under evaluation for use in biocidal products for human hygiene, within the review program of existing active substances established under Regulation (EU) No 528/2012 and as referenced in Commission Decision (EU) 2016/1950.^14^

Nanotechnology-based systems are promising tools in antimicrobial therapy, enhancing drug solubility, stability, and pharmacokinetics.^15,16^ By delivering therapeutics within nanocarriers, they enable targeted and sustained release, improved tissue distribution following *in vivo* administration, and superior antimicrobial and antibiofilm efficacy.^17,18^ Moreover, it has been reported that BKC, which is commonly found in laundry disinfectants, household cleaners, medical devices, and other everyday products, can trigger allergic skin reactions such as contact dermatitis.^19,20^ Therefore, a nanodelivery system for BKC could also be advantageous in this context, potentially allowing a reduction in the required BKC concentration and, consequently, lowering the risk of irritation. Among the currently available nanodelivery platforms, nanoemulsions - particularly nanodroplets (NDs) - have been used to deliver antimicrobial agents across a wide range of applications, including topical ophthalmic formulations for conditions such as glaucoma and ocular hypertension, ready-to-use spray disinfectants, broad-spectrum antiviral agents and endodontic treatment.^21–23^ Indeed, NDs are a particular type of nanoemulsion that offer a simple, cost-effective, and versatile platform for both therapeutic and diagnostic applications.^24^ NDs are vesicle-like nanosystems consisting of a liquid or vaporizable *core* (e.g., perfluorocarbons such as decafluoropentane [DFP]) surrounded by an outer shell (e.g., polymers, lipids) which is generally stabilized by surfactants. In this work, the active molecule BKC, due to its amphiphilic nature, is acting as a surfactant at the phase interface, allowing for the formation of stable NDs.^23^ The antimicrobial potential of NDs has been demonstrated through the incorporation of BKC. Here, we aimed to reduce the concentration of BKC used in household disinfectant sprays, by encapsulating it in BKC-loaded nanodroplets (BKC-NDs) while enhancing its antimicrobial efficacy. Co-encapsulation of limonene, a natural antimicrobial and antioxidant monocyclic monoterpene present in numerous essential oils from Citrus, resulted in a marked enhancement of the overall microbicidal efficacy of the spray disinfectant.^25^ Indeed, its antimicrobial mechanism occurs primarily by disrupting bacterial membrane integrity, involving interactions with negatively charged phosphate groups on the membrane surface, which increase permeability, induce leakage of intracellular contents, and ultimately cause structural damage.^26,27^ Moreover, limonene is frequently used as a fragrance in household cleaning products formulated with QACs; however, its application is limited by its allergenic potential,^28^ low stability,^29^ and skin sensitizer properties^30^ ascribed to its oxidation products (limonene oxide, carveol and carvone)^31^ derived from exposure to air, light, and high temperatures. To this end, encapsulation in nanosized vehicles can promote stability, reducing the oxidations and the potential skin irritation induced by limonene.^32^ Finally, encapsulation of limonene in nanocarriers can protect it from improve its low water solubility, and increase its antimicrobial activity.^33,34^

Therefore, a library of BKC-NDs was developed using varying BKC concentrations (0.008, 0.08, and 2.4% w/v), with or without a DFP *core*, to identify the minimum effective dose ensuring optimal antimicrobial activity. Importantly, the exclusion of DFP as a potential environmental contaminant aligns with green chemistry principles. This study proposes a novel, eco-friendly BKC formulation incorporating naturally derived materials to reduce chemical load and limit both toxicity and environmental impact.

## 2 Experimental Section

### 2.1 Materials

All the chemicals were purchased from Sigma-Aldrich (St. Louis, MO, USA). All chemicals utilized for HPLC analysis were analytical-grade and commercially accessible.

The bacterial strains were Gram-positive bacterium *Staphylococcus aureus* ATCC 29213, Gram-negative bacteria *Escherichia coli* ATCC 25922, and two clinical strains of *Acinetobacter baumannii* MDR. Mueller Hinton broth (MHB, Sigma-Aldrich, Spain) powder and Nutrient agar (NA, Thermo Fisher, UK) were used according to the manufacturers’ instructions. Tween 80, tryptone and sodium chloride (Sigma-Aldrich, Germany) were used to prepare the neutralizing solution for the quantitative suspension assay (0.5% v/v Tween 80, 0.1% w/v tryptone, 0.85% w/v sodium chloride, water).

### 2.2 Methods

#### 2.2.1 Preparation of BKC-NDs Disinfectant Sprays

The DFP-cored NDs disinfectant sprays were prepared starting from a refined manufacturing method.^35^ Initially, 55 μL of limonene was incorporated into 300 μL of DFP in an ice bath. Subsequently, 300 μL of an aqueous 3% w/v Tween 20 solution was added dropwise under vigorous stirring, followed by the gradual addition of the appropriate volume of BKC aqueous solution to obtain final concentrations of 0.008, 0.08, and 2.4% w/v. A pre-emulsion was formed by gradually adding ultrapure water to the mixture, continuing the stirring process. The system was then homogenized using an Ultra-Turrax homogenizer (T18 digital, IKA-Werke GmbH & Co. KG, Staufen, Germany) for 2 min at 25,000 rpm until a homogeneous nanoemulsion was achieved. The same procedure was used to prepare BKC-NDs without DFP, by simply omitting it from the formulation.

All the disinfectants were prepared and stored in spray bottles at room temperature, mimicking the real conditions of a cleaning product for the house. In Table 1 the percentage composition of BKC-NDs is reported.

**Table 1:** Percentage Composition of Optimized Spray Disinfectants

spray disinfectant	DFP (% v/v)	Tween 20 (% v/v)	BKC (% w/v)	limonene (% v/v)
BKC-NDs 0.008% w/v	5	0.16	0.008	1
BKC-NDs 0.08% w/v	5	0.16	0.08	1
BKC-NDs 2.4% w/v	5	0.16	2.4	1
BKC-NDs 0.008% w/v (w/o DFP)		0.16	0.008	1
BKC-NDs 0.08% w/v (w/o DFP)		0.16	0.08	1
BKC-NDs 2.4% w/v (w/o DFP)		0.16	2.4	1

#### 2.2.2 Physicochemical Characterization of BKC-NDs Disinfectant Sprays

The physicochemical properties of NDs and BKC-loaded-NDs were characterized, including average diameter, polydispersity index (PDI) and ζ potential (ζ) using a Dynamic Light Scattering (Zetasizer PRO, Malvern Panalytical, UK) at room temperature, after dilution in deionized water (1:100 v/v). The pH of the formulations was determined using a pH meter (model 420A, LAISS Apparecchi scientifici).

#### 2.2.3 Evaluation of the Rheological Profile of BKC-NDs Disinfectant Sprays

Rheological analyses were carried out at 25 °C using a rotational viscometer (Brookfield LVDV-III, Brookfield Engineering Laboratories, Middleboro, MA, USA) with an SC4–18 spindle, maintained at constant temperature via a water bath. BKC-NDs spray formulations were tested by loading 8 mL of sample into the measurement chamber. Viscosity and shear stress were recorded across shear rates from 66 to 330 s^–1^. Each test was performed in triplicate to assess reproducibility.

#### 2.2.4 Measurement of Surface Tension in BKC-NDs Disinfectant Sprays

The γ measurement of BKC-ND formulations was assessed using a digital tensiometer in triplicate (Krüss GmbH K10 PST, Hamburg, Germany). The γ values were compared to those of pure water and an aqueous BKC solution at equivalent concentration.

#### 2.2.5 Evaluation of the Stability of BKC-NDs Disinfectant Sprays Under Various Storage Conditions

Stability was assessed by monitoring changes in color, pH, the presence of precipitation or phase separation, and variations in average particle diameter. The chemical and physical stability of the BKC-NDs disinfectants was evaluated under different environmental conditions, exposed to light, including room temperature, 37 °C, and accelerated aging conditions (45–50 °C), to simulate long-term storage.

#### 2.2.6 Spray Angle Measurement as an Indicator of Disinfectant Coverage Efficiency

The spray angles of BKC-NDs disinfectants were measured after adding two drops of a 1% w/v eosin aqueous solution to enable visualization. The disinfectants were sprayed onto blotting paper while maintaining the bottle in a horizontal position at a fixed distance of 15 cm between the nozzle and the application surface. The spray angle (*q*) was calculated using eq 1:

1 $$\theta =2\;\tan^{-1}\left(\frac{d}{2L}\right)$$

where *d* is the diameter of the spray pattern on the blotting paper and *L* is the distance from the nozzle to the surface.

#### 2.2.7 Evaluation of Surface Wettability of BKC-NDs Disinfectant Sprays Using Contact Angle Analysis

The surface wettability of BKC-NDs on solid surfaces such as glass or plastic was evaluated via contact angle measurement using an optical tensiometer (Attension Theta Lite, Biolin Scientific). The contact angle was measured by depositing a droplet of liquid on the desired substrate, and its profile was recorded with a high-resolution camera, and analyzed with the Young–Laplace model. Angles under 90° were considered indicative of good wettability and efficient surface coverage of the disinfectants.^36^

#### 2.2.8 Encapsulation Efficiency and Loading Capacity of BKC in BKC-NDs Disinfectant Sprays

The encapsulation efficiency (EE%) and loading capacity (LC%) of BKC-NDs were determined using a validated HPLC method with eqs 2 and 3:

2 $$\mathrm{EE}\%=\frac{\mathrm{total}\;\mathrm{drug}-\mathrm{free}\;\mathrm{drug}}{\mathrm{total}\;\mathrm{drug}}\times 100\%$$

3 $$\mathrm{LC}\%=\frac{\mathrm{amount}\;\mathrm{of}\;\mathrm{drug}\;\mathrm{in}\;\mathrm{NB}}{\mathrm{freeze‐dried}\;\mathrm{NB}\;\mathrm{weight}}\times 100\%$$

Analyses were performed on a Shimadzu system equipped with a UV/vis spectrophotometric detector and a Thermo Scientific Acclaim Surfactant Plus column (150 × 3 mm, 3 μm), which operates based on a mixed-mode separation mechanism. The mobile phase (ACN/75 mM KH_2_PO_4_, pH 3, 1:1 v/v) was pumped at 0.425 mL/min. Samples were diluted (ACN/water, 1:1 v/v), filtered (0.22 μm), and injected (20 μL) at 25 °C.

#### 2.2.9 *In Vitro* Release Studies of BKC from the BKC-NDs Disinfectant Sprays

BKC *in vitro* release from BKC-NDs was assessed using a dialysis method. Each 3 mL sample was sealed in a 14 kDa cutoff cellulose membrane (Spectra/Por) and immersed in 30 mL of water. At set intervals over 24 h, 1 mL of the medium was replaced with fresh water to maintain sink conditions. Samples were analyzed via HPLC, and release profiles were plotted over time.

#### 2.2.10 Antimicrobial Assay of BKC-NDs Disinfectant Sprays

MIC and MBC were assessed by a 2-fold serial dilution method in 96-well plates, with disinfectant concentrations ranging from 400 to 0.19 (formulation at 0.08% w/v). Each well received 100 μL of bacterial suspension (10^6^ CFU/mL) and was incubated at 37 °C for 24 h. MIC was defined as the lowest concentration with no visible growth; the MBC was assessed using the MIC concentration and the two concentrations over the MIC, to be subcultured on Nutrient agar plates, and incubated overnight at 37 °C. MBC as the lowest concentration, showing no CFUs upon subculturing. Untreated bacteria served as controls. Tests were performed in duplicate and repeated at least three times.

#### 2.2.11 Quantitative Suspension Test Method (EN 1040:2005/13727:2015/1276:2019)

The quantitative suspension test was conducted in accordance with the EN 1040:2005/13727:2015/1276:2019 standards, which outlines the test methods and requirements for evaluating the bactericidal activity of chemical disinfectants and antiseptics.^37–39^ Unless otherwise specified, the tests were performed at 30 °C, selected to simulate slightly more demanding, realistic conditions, particularly relevant in medical and hospital environments, where ambient or surface temperatures may exceed the standard 20–25 °C range. Additional experiments at 20 °C were conducted to assess the influence of temperature on antimicrobial efficacy. A chemical disinfectant meets the standards when it achieves a 5-log reduction in the bacterial cell count (CFU/mL) of the tested strain. According to regulatory guidelines, the required reduction factor (RF) for antiseptic or disinfectant agents ranges from ≥3 to ≥5, depending on the specific application area.^40^

Bacterial cultures were grown in Mueller-Hinton broth for 18–24 h and diluted in sterile isotonic NaCl to 10^8^ CFU/mL. To mimic contamination with organic compounds, such as in wound or skin conditions, a parallel inoculum was prepared with bovine serum albumin (BSA, 0.3 mg/mL), following EN 1040:2005/13727:2015/1276:2019 guidelines.

Each inoculum (0.1 mL) was mixed with 0.4 mL of the test formulation and incubated at 20 or 30 °C at different time points as indicated in the Results section. The reaction was neutralized by adding 0.05 mL sterile water and 0.4 mL of neutralizing solution, followed by 5 min in a 20 °C water bath. Serial dilutions were plated on Nutrient agar, and CFUs were counted after 24 h at 37 °C.

To evaluate the synergistic action of limonene in BKC-NDs, the same test method was performed starting from 10^9^ CFU/mL of *E. coli* with the following test formulations: BKC-NDs with or without 1% limonene, NDs 1% limonene, free 1% limonene and free BKC.

The bactericidal effect was expressed as the reduction factor (RF), which is given by eq 4:

4 $$\mathrm{RF}=\log_{10}\left(\mathrm{nc}\right)-\log_{10}\left(\mathrm{nd}\right)$$

where nc and nd are CFUs before and after treatment, respectively. Tests were performed in duplicate and repeated in triplicate.

#### 2.2.12 Interaction of NDs with Bacteria by Confocal Laser Microscopy

To evaluate the interaction of prepared NDs with *E. coli* (10^7^ CFU/mL), bacteria were incubated for 15 min at room temperature under gentle agitation in the absence or presence of 10% v/v blank NDs previously labeled with coumarin-6 (Sigma-Aldrich). Following incubation, the bacterial suspension was centrifuged at 3000*g* for 10 min at room temperature and washed twice with 1× PBS to remove unbound NDs. The final pellet was resuspended in 0.5 mL of PBS, and 50 μL of the suspension was transferred onto glass slides, heat-fixed, and stained with 5 μg/mL propidium iodide (Merck Life Science S.r.l., Italy) for 15 min in a humid chamber at 37 °C. Samples were then mounted with mounting medium and covered with coverslips. Slides were imaged using a ZEISS LSM 800 microscope equipped with a 63× oil-immersion objective using Airyscan confocal super-resolution and default Airyscan acquisition settings. Images were further processed with the deconvolution-based Airyscan processing tool in ZEN 3.0 software to enhance spatial resolution. Image analysis and final figure preparation were performed using ImageJ/FIJI.^41^

#### 2.2.13 Cytotoxicity Assay

The biocompatibility of NDs was tested using Human Foreskin Fibroblasts (HFF) cells. Cells were seeded at 0.1 × 10^6^ cells/ml in 12-well plates and incubated overnight at 37 °C and 5% CO_2_ in DMEM supplemented with 10% FBS, 2 mM glutamine, and 1% antibiotics (Gibco, Thermo Fisher Scientific, Waltham, MA, USA). After incubation, cells were exposed for 24 h to BKC-NDs or free BKC at different concentrations. Cell viability was assessed using the MTT assay (Merk Life Science S.r.l, Italy). All conditions were tested in triplicate, and absorbance was measured at 570 nm using a microplate reader (VICTOR3TM, PerkinElmer, CT, United States).

## 3 Results and Discussion

A library of BKC-NDs with and without a *core* of DFP was successfully developed with three different BKC concentrations: a low BKC concentration (0.008% w/v), an intermediate BKC concentration (0.08% w/v), and a high BKC concentration (2.4% w/v). All formulations exhibited excellent encapsulation efficiency (EE > 99%), confirming the effectiveness of the nanoemulsion strategy in incorporating and stabilizing BKC within BKC-NDs. As expected, increasing the BKC concentration resulted in a higher loading capacity (LC%) for both formulations with and without DFP. Furthermore, the calculated BKC concentration in the formulations confirmed efficient incorporation of the surfactant into the NDs (Table 2). Limonene was successfully incorporated in BKC-NDs with EE% values above 90% for all the developed NDs (Table S1 and Figure S2).

**Table 2:** Encapsulation Efficiency (EE%) and Loading Capacity (LC%) of BKC in BKC-NDs^a^

formulation	EE%	LC%	BKC conc. (mg/mL)
BKC-NDs 0.008% w/v	99.00 ± 2.00	0.82 ± 0.03	0.08 ± 0.0005
BKC-NDs 0.08% w/v	94.00 ± 1.00	7.19 ± 0.31	0.78 ± 0.01
BKC-NDs 2.4% w/v	93.00 ± 7.00	67.22 ± 5.19	24.55 ± 0.18
BKC-NDs 0.008% w/v (w/o DFP)	97.50 ± 1.10	0.75 ± 0.01	0.08 ± 0.0006
BKC-NDs 0.08% w/v (w/o DFP)	99.17 ± 1.71	7.18 ± 0.11	0.82 ± 0.02
BKC-NDs 2.4% w/v (w/o DFP)	90.80 ± 0.37	70.29 ± 0.08	25.16 ± 0.96

a Data are expressed as mean ± SD.

**Table 3:** Physicochemical Parameters of BKC-NDs^a^

ND formulation	*D*_avg_ (nm)	PDI	ζ (mV)	pH
NDs blank	540.23 ± 68	0.39 ± 0.01	–12.01 ± 0.59	5.10
BKC-NDs 0.008% w/v	791.12 ± 44	0.30 ± 0.01	+32.76 ± 0.58	5.50
BKC-NDs 0.08% w/v	273.00 ± 14	0.28 ± 0.04	+65.47 ± 1.05	4.40
BKC-NDs 2.4% w/v	119.12 ± 1.26	0.17 ± 0.03	+73.67 ± 2.98	4.50
BKC-NDs 0.008% w/v (w/o DFP)	252.12 ± 16	0.20 ± 0.06	+4.91 ± 1.11	5.82
BKC-NDs 0.08% w/v (w/o DFP)	226.00 ± 1.66	0.24 ± 0.01	+11.73 ± 2.00	4.50
BKC-NDs 2.4% w/v (w/o DFP)	243.13 ± 23	0.21 ± 0.02	+15.44 ± 1.25	5.33

a *D*_avg_ is the average diameter; PDI is the polydispersity index; ζ is the ζ potential. Data are expressed as mean ± SD.

The BKC-NDs spray disinfectants exhibited a nanoscale diameter and a positive surface charge attributable to the presence of the BKC coating (Table 3). The mean diameter of the NDs increased following BKC incorporation, rising from approximately 540 nm for the blank NDs—prepared without BKC—to about 791 nm when loaded with 0.008% w/v BKC. As the BKC concentration increased, the average diameter of the BKC-NDs decreased. This effect is attributed to the BKC concentration exceeding its Critical Micelle Concentration (CMC), calculated to be 1.19 mg/mL (Figure S1). Beyond this threshold, surfactant molecules spontaneously self-assemble to minimize interfacial tension, leading to the formation of micelles—more stable colloidal dispersions with smaller and more uniform particle sizes. The average micelle size was approximately 100 nm, leading to BKC-NDs with 2.4% w/v BKC showing a reduced average diameter of about 118 nm. In the absence of DFP, BKC-NDs without DFP exhibited a smaller average diameter compared to their counterparts containing DFP at the same concentration.

The BKC-NDs containing DFP exhibited a high ζ potential values (from +32.76 mV for NDs with 0.008% w/v BKC up to +73.67 mV for those with 2.4% w/v BKC), whereas the corresponding formulations prepared without DFP, containing solely limonene in the organic phase, showed a marked reduction in surface charge (from +4.91 mV for NDs with 0.008% w/v BKC up to +15.44 mV for those with 2.4% w/v BKC). When DFP is present in the *core*, BKC intercalates at the interface, with its hydrophilic portion completely oriented toward the aqueous phase, resulting in a higher positive surface charge. In contrast, in BKC-NDs without DFP (where only limonene is present in the *core*), the interfacial packing of BKC presents a different distribution, leading to a substantial decrease in ζ potential. Consistently, the blank NDs displayed a negative ζ potential, as DFP remains stably entrapped in the *core* and Tween 20 (polysorbate 20) occupies the interfacial layer. Moreover, the ζ potential of the formulations increased proportionally with the BKC concentration, confirming the presence of BKC at the interface.^23^

The pH values of all formulations remained relatively stable, ranging between 4.5 and 5.5.

As shown by surface tension and contact angle measurements, BKC-NDs exhibited enhanced interfacial activity and wettability compared to aqueous BKC solutions, interestingly even at low concentrations. Overall, at BKC concentrations (0.008% w/v, 0.08 mg/mL) below the CMC, aqueous BKC solutions did not significantly reduce surface tension compared to that of pure water (∼70 mN/m), confirming minimal surfactant activity. In contrast, all disinfectants formulated as BKC-NDs exhibited a pronounced reduction in surface tension even at low concentrations, attributed to the presence of Tween 20 and to the improved surfactant distribution enabled by the nanoemulsion system (Figure 1).

**Figure 1:**
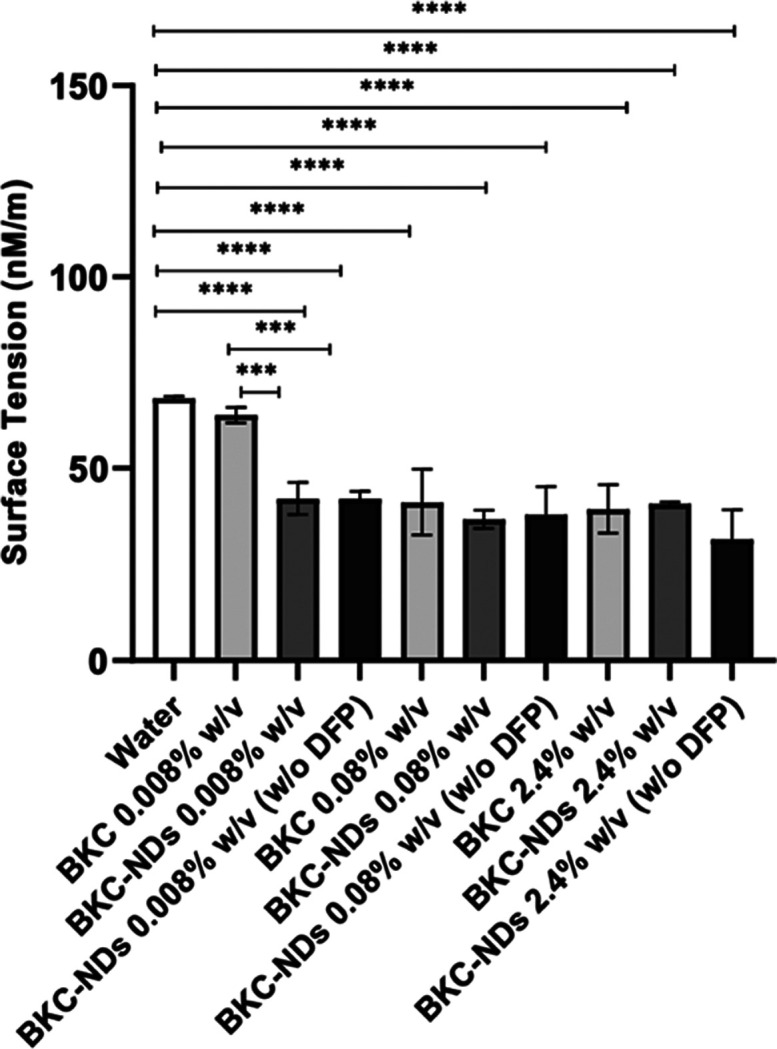
Comparison of surface tension between BKC-NDs, BKC aqueous solution and water. Data are expressed as mean ± SD. One-way ANOVA followed by Sidak’s multiple comparison test showed statistical significance between water and BKC-NDs (***p < 0.001, ****p < 0.0001), and between BKC 0.008% w/v and BKC-NDs 0.008% w/v (**p < 0.01).

Furthermore, BKC-NDs displayed lower contact angles than aqueous BKC solutions, confirming their improved surface wettability (Table 4, contact angle images are reported in Figure S3). At increasing BKC concentration a decrease in contact angle was observed, indicating enhanced wettability of the solution, particularly near and above CMC, at 0.08% w/v (0.8 mg/mL) and 2.4% w/v (24 mg/mL), where micelle formation occurs. Together, the reduced surface tension and lower contact angle observed for BKC-NDs indicate enhanced wettability and spreading behavior, which may ultimately contribute to improved detergent and disinfectant performance compared to standard BKC solutions.

**Table 4:** Contact Angle Measurements of BKC-NDs on Different Surfaces Mimic Various Cleaning Scenarios, Respectively on Glass Surface and Plastic Surface

	contact angle (deg)	
formulation	glass surface	plastic surface
water	32	36
BKC 0.008% w/v	55	67
BKC 0.08% w/v	37	47
BKC 2.4% w/v	20	37
BKC-NDs 0.008% w/v	24	38
BKC-NDs 0.08% w/v	8	28
BKC-NDs 2.4% w/v	12	11
BKC 0.008% w/v (w/o DFP)	23	27
BKC 0.08% w/v (w/o DFP)	9	9
BKC 2.4% w/v (w/o DFP)	11	5

From the rheological point of view BKC-NDs exhibited a typical Newtonian fluid behavior, characterized by a constant viscosity as the shear rate increased, that enables easy and uniform spreading and contributes to long-term stability (Figure 2). The low slope of the flow curve indicates that BKC-NDs behave as Newtonian fluids with a viscosity comparable to that of pure water.

**Figure 2:**
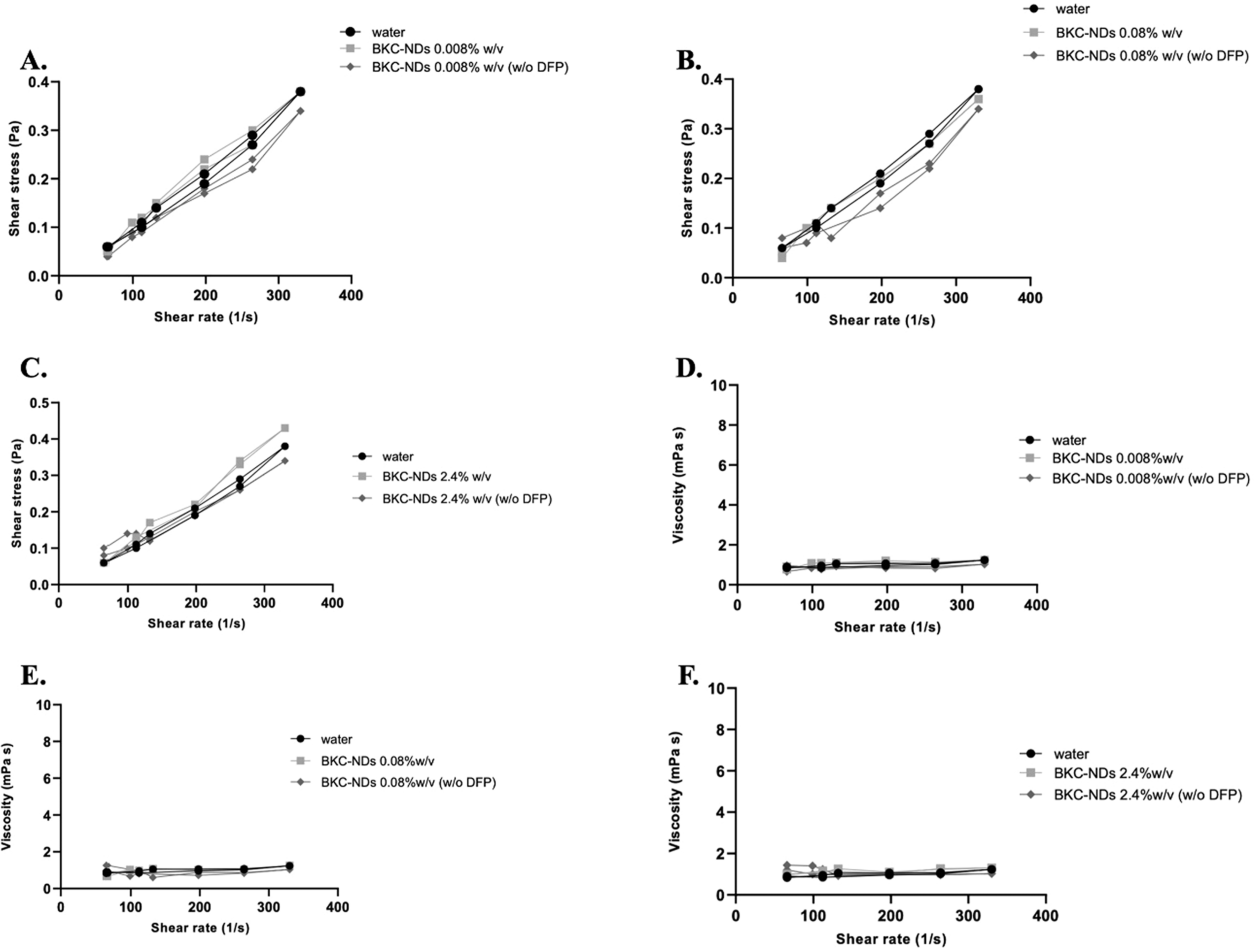
Shear stress vs shear rate curve of BKC-NDs with and without DFP at 0.008% w/v (A), 0.08% w/v (B), 2.4 w/v (C); Viscosity vs shear rate curve of BKC-NDs with and without DFP at 0.008% w/v (D), 0.08% w/v (E) and 2.4% w/v (F) compared to pure water.

The formulation of BKC as BKC-NDs significantly improved the liquid dispersion of the disinfectants after spraying, thereby enhancing surface coverage and overall efficiency. In particular, at concentrations of 0.008 and 0.08% w/v, spray angles reached values of up to ∼95°, ensuring broader and more uniform surface coverage. In comparison, pure water exhibited a spray angle of approximately 62°, while aqueous BKC solutions showed smaller angles ranging from 42 to 48°, as reported in Table 5. However, at a higher concentration of 2.4% w/v, above the CMC value, micelle formation led to a reduction in spray angle, thereby limiting dispersion. These findings indicate that the addition of surfactants and the nanoemulsion structure of BKC-NDs significantly enhance liquid spreading, ultimately improving the application and effectiveness of the disinfectant.

**Table 5:** Spray Angles Measurements of BKC-NDs Were Wider than Water and BKC Aqueous Solutions

formulation	spray angle (deg)
water	62
BKC 0.008% w/v	44
BKC 0.08% w/v	49
BKC 2.4% w/v	42
BKC-NDs 0.008% w/v	96
BKC-NDs 0.08% w/v	86
BKC-NDs 2.4% w/v	69
BKC 0.008% w/v (w/o DFP)	88
BKC 0.08% w/v (w/o DFP)	84
BKC 2.4% w/v (w/o DFP)	47

Furthermore, the formulations demonstrated long-term stability over six months, with no evidence of phase separation, pH and/or color changes, or precipitation throughout the observation period. The variation in the average diameter of BKC-NDs was monitored over time under different environmental conditions (room temperature, 45 °C). At room temperature, a gradual decrease in average diameter was observed over time, as shown in Figure 4 panel A, likely due to micelle reorganization, with sizes reaching just a few nanometers after six months. Increasing the storage temperature up to 45 °C resulted in a more pronounced reduction in the average diameter over time, as observed at 1 month and 6 months, and shown in Figure 3 panel B.

**Figure 3:**
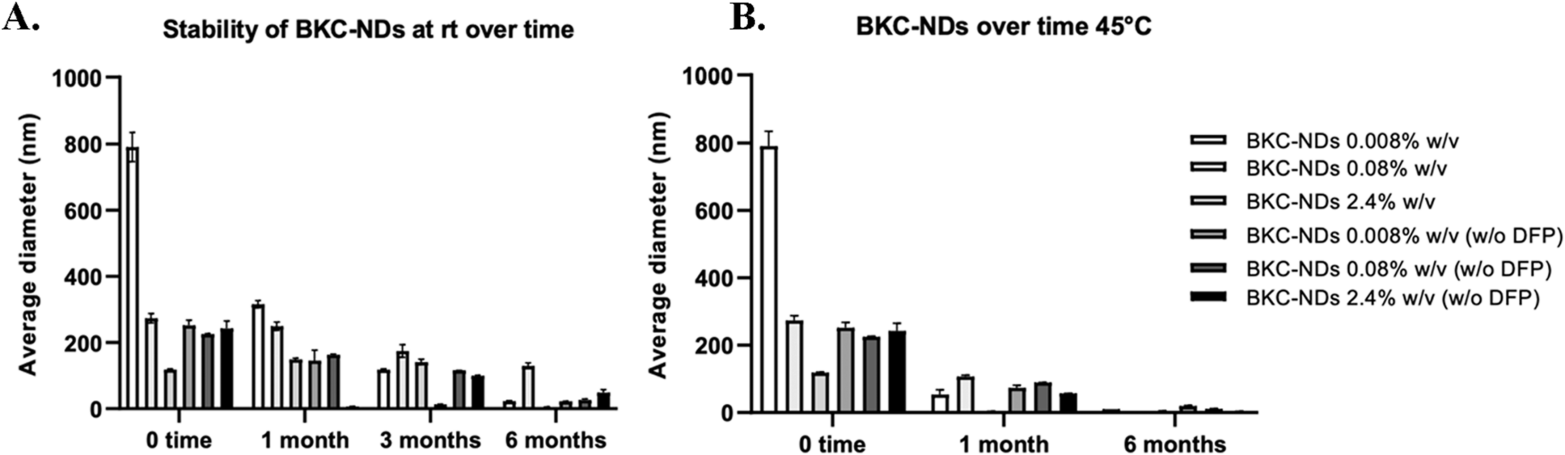
Stability of the disinfectant over time at different environmental conditions: (A) over a period of 6 months at rt; (B) over 6 months at 45 °C. Bars represent mean ± SD (*n* = 3). Statistical analysis was performed using two-way ANOVA with Factor A (Temperature or Time) and Factor B (Average diameter). A significant main effect was found for Factor A (*p* < 0.0001), Factor B (*p* < 0.0001) and their interaction (*p* < 0.0001). Post hoc comparison (Tukey’s HSD) revealed significant differences between selected groups.

**Figure 4:**
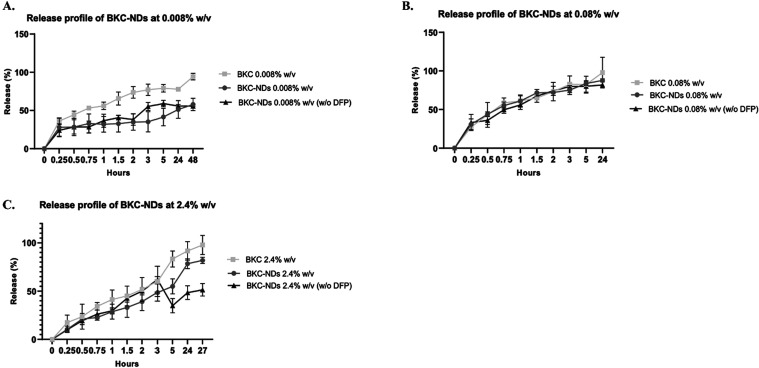
BKC release profile of formulations at 0.008, 0.08 and 2.4% w/v from BKC-NDs compared to that at the same BKC concentration in aqueous solution. Data are expressed as mean ± SD. Statistical analysis performed using Two-way ANOVA followed by Sidak’s multiple comparison test: both time and formulation showed (A) time and formulation showed significant effect (****p < 0.0001). (B) time and formulation showed no significant effect (ns). (C) time, formulation and their interaction showed a significant effect (***p < 0.0001).

The formulation of BKC as BKC-NDs maintains the ability to release the active compound immediately, similarly to the aqueous BKC formulation (Figure 4). Notably, the release profile of BKC from the 0.08% w/v BKC-NDs exhibits an initial burst release, which correlates with the higher antimicrobial activity observed compared to the 0.008% w/v formulation, where the lower concentration resulted in a more sustained release pattern. In contrast, at the highest tested concentration (2.4% w/v), BKC release was even more prolonged, likely due to micelle formation, which reduces the amount of free BKC available for immediate release and may consequently lower the initial antimicrobial efficacy.

The antimicrobial efficacy of the 0.08% w/v BKC-NDs formulation was confirmed through MIC and MBC determinations (Table 6). Against *S. aureus* ATCC 29293, the MIC value decreased from 1.56 μg/mL (aqueous BKC) to 0.78 μg/mL in BKC-NDs. Similarly, for *E. coli* ATCC 25922, the MIC was reduced from 6.25 to 3.125 μg/mL.

**Table 6:** MIC and MBC Values of BKC-NDs at 0.08% w/v, with and without DFP, Against *S. aureus*, *E. coli*, and *A. baumannii* MDR

***S. aureus* ATCC 29293**		
formulation	MIC BKC (% w.r.t. starting solution)	MBC
BKC-NDs 0.08% w/v	0.78 μg/mL (0.094%)	0.78 μg/mL
BKC-NDs 0.08% w/v (w/o DFP)	0.78 μg/mL (0.094%)	1.5 μg/mL
BKC	1.5 μg/mL	1.5 μg/mL
blank	>50%	>50%
blank + 1% limonene	>50%	>50%

***E. coli* ATCC 25922**		
formulation	MIC BKC (% w.r.t. starting solution)	MBC
BKC-NDs 0.08% w/v	3.125 μg/mL (0.38%)	6.25 μg/mL
BKC-NDs 0.08% w/v (w/o DFP)	3.125 μg/mL (0.38%)	6.25 μg/mL
BKC	6.25 μg/mL	6.25 μg/mL
blank	>50%	>50%
blank + 1% limonene	50%	>50%

***A. baumannii* MDR**		
formulation	MIC BKC (% w.r.t. starting solution)	MBC
BKC-NDs 0.08% w/v	6.25 μg/mL (0.75%)	12.5 μg/mL
BKC-NDs 0.08% w/v (w/o DFP)	6.25–12.5 μg/mL	12.5 μg/mL
BKC	12.5–24 μg/mL	12.5–24 μg/mL
blank	>50%	>50%
blank + 1% limonene	>50%	>50%

Further evaluations were conducted against two multidrug-resistant strains of *A. baumannii* where an enhanced antimicrobial activity was observed (from 12.5–24 μg/mL for free BKC to 6.25–12.5 μg/mL for BKC-NDs), confirming its ability to improve BKC effectiveness even against resistant bacterial strains. The MBC values were similar to those obtained with free BKC, except that for *S. aureus* and *A. baumannii*, the values obtained with BKC-NDs were lower than those obtained with free BKC. Interestingly, blank NDs with 1% limonene showed a minimal antimicrobial activity against *E. coli* (MIC at 50% of the concentration concerning the starting solution), indicating a potential synergistic interaction when coloaded with BKC (Tables 6 and 7).

**Table 7:** Reduction as Log_10_ of Bacterial Cell Count (log CFU/mL) (RF) Evaluated Performing the ISO EN 1040:2005/13727:2015/1276:2019 Against *S. aureus* and *E. coli* to Determine the Basic Bactericidal Activity of Chemical Disinfectants and Antiseptics under the Tested Conditions (15 min, 1 h, and 6 h)^a^

	RF at 15 min		RF at 1 h		RF at 6 h	
formulation	–BSA	+BSA	–BSA	+BSA	–BSA	+BSA
***S. aureus***						
Blank NDs + 1% limonene	0.05 ± 0.08	–0.003 ± 0.006	0.69 ± 0.98	0.13 ± 0.08	3.79 ± 2.17	1.27 ± 1.30
BKC 0.008% w/v	–0.06 ± 0.18	0.05 ± 0.1	0 ± 0.05	0.017 ± 0.10	0.025 ± 0.29	0.46 ± 1.06
BKC-NDs 0.008% w/v	3.32 ± 0.34	3.43 ± 1.25	3.82 ± 1.43	3.63 ± 1.56	3.92 ± 1.40	4.79 ± 0.91
BKC-NDs 0.008% w/v w/o DFP	3.11 ± 0.68	4.07 ± 1.58	2.26 ± 0.57	4.42 ± 1.76	4.19 ± 1.32	4.46 ± 1.47
BKC 0.08% w/v	2.35 ± 3.06	3.35 ± 0.69	3.97 ± 2.12	3.87 ± 1.96	4.85 ± 1.01	5.34 ± 0.07
BKC-NDs 0.08% w/v	4.71 ± 0.73	5.03 ± 0.08	5.39 ± 0.09	4.84 ± 1.05	5.26 ± 0.22	5.34 ± 0.07
BKC-NDs 0.08% w/v w/o DFP	5.24 ± 0.16	5.03 ± 0.08	5.40 ± 0.10	5.40 ± 0.15	5.33 ± 0.20	5.34 ± 0.07
BKC 2.4% w/v	4.74 ± 0.71	4.44 ± 0.07	5.46 ± 0.16	4.84 ± 1.05	5.26 ± 0,22	5.34 ± 0.07
BKC-NDs 2.4% w/v	6.24 ± 0.16	5.03 ± 0.08	5.39 ± 0.09	5.40 ± 0.15	5.26 ± 0.22	5.34 ± 0.07
BKC-NDs 2.4% w/v w/o DFP	6.24 ± 0.16	5.03 ± 0.08	5.40 ± 0.10	5.40 ± 0.15	5.33 ± 0.20	5.34 ± 0.07
log_10_ of CFUs in the inoculum (control w/o treatment)	5.24 ± 0.16	5.03 ± 0.08	5.46 ± 0.13	5.40 ± 0.15	5.33 ± 0.26	5.34 ± 0.07
***E. coli***						
Blank NDs + 1% limonene	4.79 ± 0.87	4.58 ± 0.82	5.405 ± 0.11	4.99 ± 1.02	4.37 ± 1.19	4.48 ± 1.79
BKC 0.008% w/v	0.14 ± 0.12	0.16 ± 0.32	1.35 ± 2.43	0.19 ± 0.09	0.31 ± 0.57	2.24 ± 3.60
BKC-NDs 0.008% w/v	5.03 ± 0.47	5.03 ± 0.48	5.41 ± 0.11	5.56 ± 0.10	4.96 ± 1.00	5.62 ± 0.76
BKC-NDs 0.008% w/v w/o DFP	5.13 ± 0.56	5.03 ± 0.48	476 ± 1.10	4.09 ± 1.34	5.35 ± 0.22	5.85 ± 0.48
BKC 0.08% w/v	3.38 ± 1.76	4.7 ± 1.05	4.904 ± 1.18	4.81 ± 1.66	4.33 ± 2.24	5.42 ± 0.97
BKC-NDs 0.08% w/v	5.03 ± 0.47	5.03 ± 0.48	4.98 ± 0.79	5.56 ± 0.10	5.38 ± 0.19	5.38 ± 0.88
BKC-NDs 0.08% w/v w/o DFP	5.03 ± 0.47	5.03 ± 0.48	5.43 ± 0.13	5.56 ± 0.10	5.35 ± 0.22	5.28 ± 1.28
BKC 2.4% w/v	5.03 ± 0.47	5.03 ± 0.48	5.43 ± 0.13	5.57 ± 0.09	5.09 ± 0.46	5.85 ± 0.48
BKC-NDs 2.4% w/v	5.03 ± 0.47	5.03 ± 0.48	4.74 ± 1.42	5.57 ± 0.09	5.38 ± 0.19	5.85 ± 0.48
BKC-NDs 2.4% w/v w/o DFP	5.03 ± 0.47	5.03 ± 0.48	5.43 ± 0.13	4.67 ± 1.47	5.39 ± 0.30	5.85 ± 0.48
log_10_ of CFUs in the inoculum (control w/o treatment)	5.03 ± 0.47	5.03 ± 0.48	5.43 ± 0.13	5.57 ± 0.09	5.38 ± 0.19	5.85 ± 0.48

a RFs of *S. aureus* and *E. coli* colony-forming units (mean ± SD of three independent experiments).

To investigate long-term stability, the antimicrobial activity of BKC-NDs was examined at 6 months postpreparation. Upon aging, the nanoformulation (BKC-NDs 0.08% w/v) rearranges into smaller and more ordered micellar structures,^42,43^ which modulates the accessibility of BKC and limonene. Gram-positive *S. aureus*, lacking an outer membrane, remains susceptible to the synergistic membrane-disrupting effect of cationic micelles comprising BKC, Tween 20 and limonene, maintaining a MIC lower than free BKC (0.78 μg/mL for BKC-NDs 0.08% w/v, vs 1.5 μg/mL for free BKC) even after 6 months; in contrast MIC of *E. coli* returns to the same value as free BKC (6.25 μg/mL BKC-NDs 0.08% w/v and free BKC). Gram-negative bacteria such as *E. coli* possess an outer membrane that limits penetration of micelle-associated BKC, Tween 20 and limonene, similarly to the free BKC. This indicates a structure-dependent differential activity driven by bacterial envelope architecture and the altered bioavailability of BKC after micelle formation.^44^

To further evaluate the efficacy of nanoformulations an *in vitro* quantitative suspension test was performed. BKC-NDs disinfectant sprays demonstrated effective basic bactericidal activity against the tested strains, according to the EN 1040:2005/13727:2015**/**1276:2019 (Table 7).

Indeed, under the tested conditions (15 min, 1 h, and 6 h at 30 °C), BKC-NDs disinfectant sprays achieved a ≥5-log reduction in *S. aureus* from a concentration as low as 0.08%, indicating rapid and sustained bactericidal activity. In contrast, the same concentration of BKC in aqueous solution yielded a significantly lower reduction (2.35 ± 3.06) after 15 min, highlighting the enhanced efficacy provided by nanoencapsulation. After 1 h, the performance of 0.08% BKC-NDs matched that of 2.4% BKC solution, confirming the potential of nanoformulations to reduce the required active concentration. Against *E. coli*, BKC-NDs were even more effective, reaching a 5-log reduction at just 0.008% in 15 min. The presence of organic matter, simulated with bovine serum albumin (BSA), did not significantly impact the antimicrobial activity. Figures 5 and 6 illustrate the time-dependent bacterial reduction for both strains compared to untreated bacteria (CTRL).

**Figure 5:**
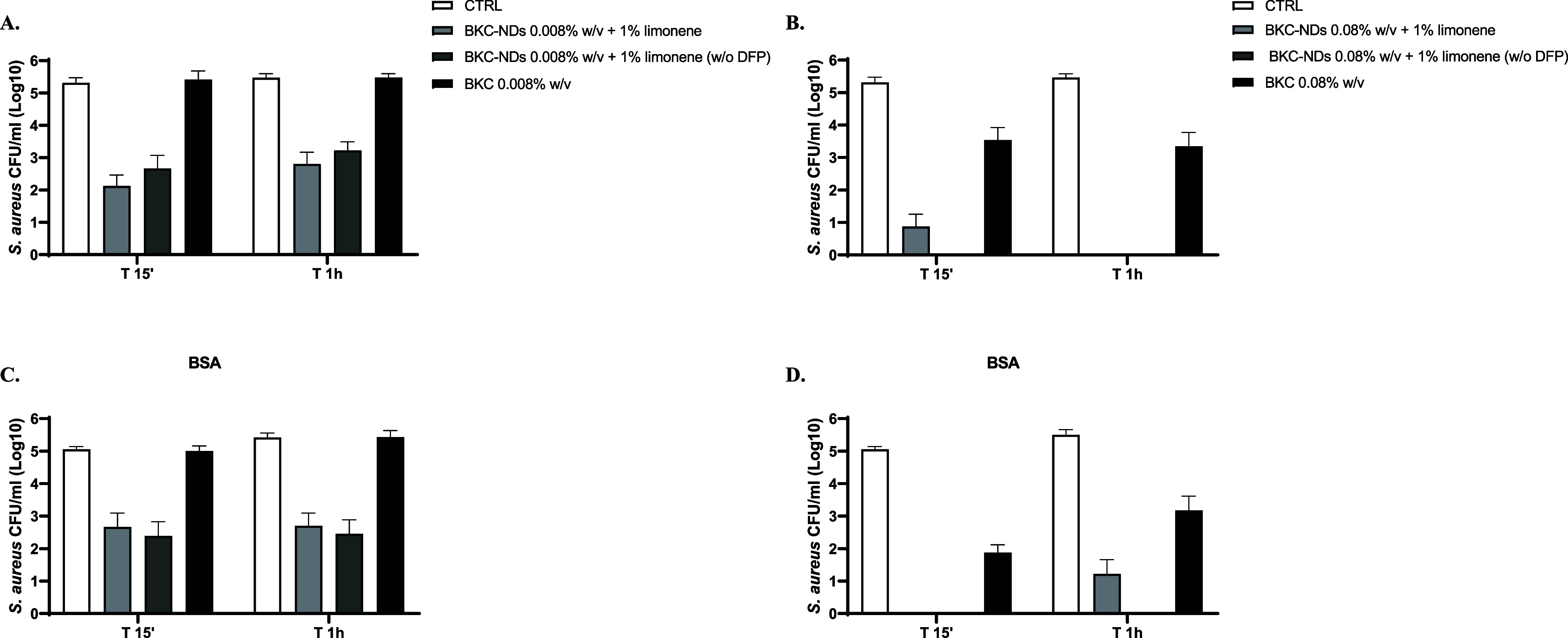
Reduction of *S. aureus* expressed as Log_10_ of CFU/mL in the presence of BKC-NDs at 0.008 and 0.08% w/v, without or with BSA (in panels A, B and C, D, respectively). Data are expressed as mean ± SD of three independent experiments.

**Figure 6:**
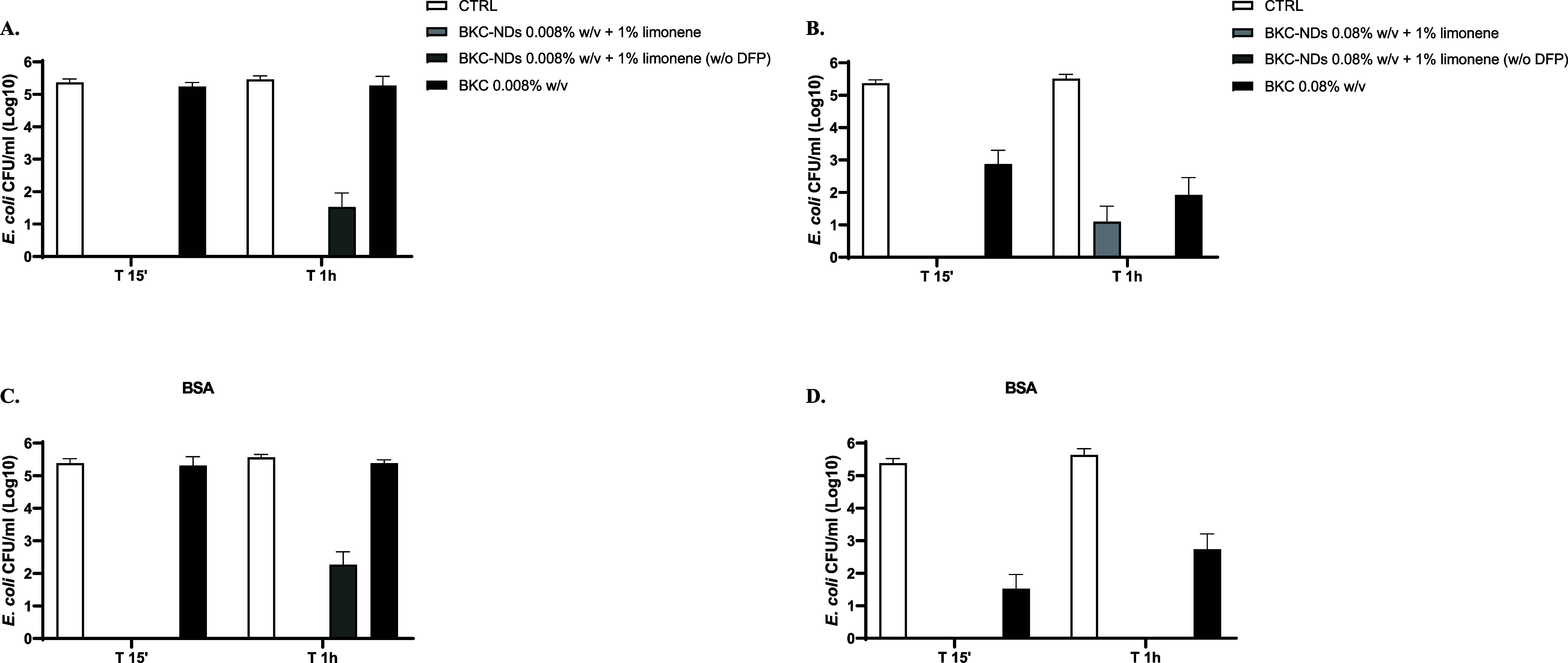
Reduction of *E. coli* expressed as Log_10_ of CFU/mL in the presence of BKC-NDs at 0.008 and 0.08% w/v, without or with BSA (in panels A, B and C, D, respectively). Data are expressed as mean ± SD of three independent experiments.

Moreover, short contact times (30 s and 5 min) were sufficient to induce a clear bactericidal effect of the 0.08% BKC-NDs formulation against both tested strains, confirming its fast-acting antimicrobial performance (Figure S4).

The bactericidal activity of 0.08% BKC-NDs was also evaluated at 20 °C to assess the effect of temperature. As shown in Figure S5, comparable log-reduction levels were observed at 20 and 30 °C for all tested microorganisms, indicating that the antimicrobial efficacy of the formulation is not significantly affected within this temperature range (Figure S4).

Additionally, the blank nanoemulsion with 1% limonene (NDs 1% limonene) reduced *E. coli* by 4.79 ± 0.87 logs after 15 min, suggesting an intrinsic activity of limonene. The coencapsulation of BKC and limonene likely led to synergistic effects, further enhancing efficacy. To demonstrate the limonene-enhancing effect on BKC-NDs, a suspension test with BKC-NDs with DFP, with and without limonene, blank NDs with 1% limonene, and free limonene and BKC was performed on *E. coli* bacteria. As shown in Figure 7, Panel A, the incorporation of 1% limonene into BKC-loaded nanodroplets enhanced killing, yielding an additional log reduction of bacteria compared to BKC-NDs alone (at 0.008% w/v). This reduction in CFU achieved by coloading limonene demonstrates that limonene acts as an effective adjuvant for *E. coli*, likely by increasing outer membrane permeability, thereby facilitating BKC access to the inner membrane. This synergistic contribution was not detectable at higher BKC concentrations (0.08% w/v) because all BKC-containing formulations achieved complete inactivation, reaching the assay’s detection limit. Free limonene exhibited partial activity, similar to free BKC at 0.08% while blank NDs were inactive.

**Figure 7:**
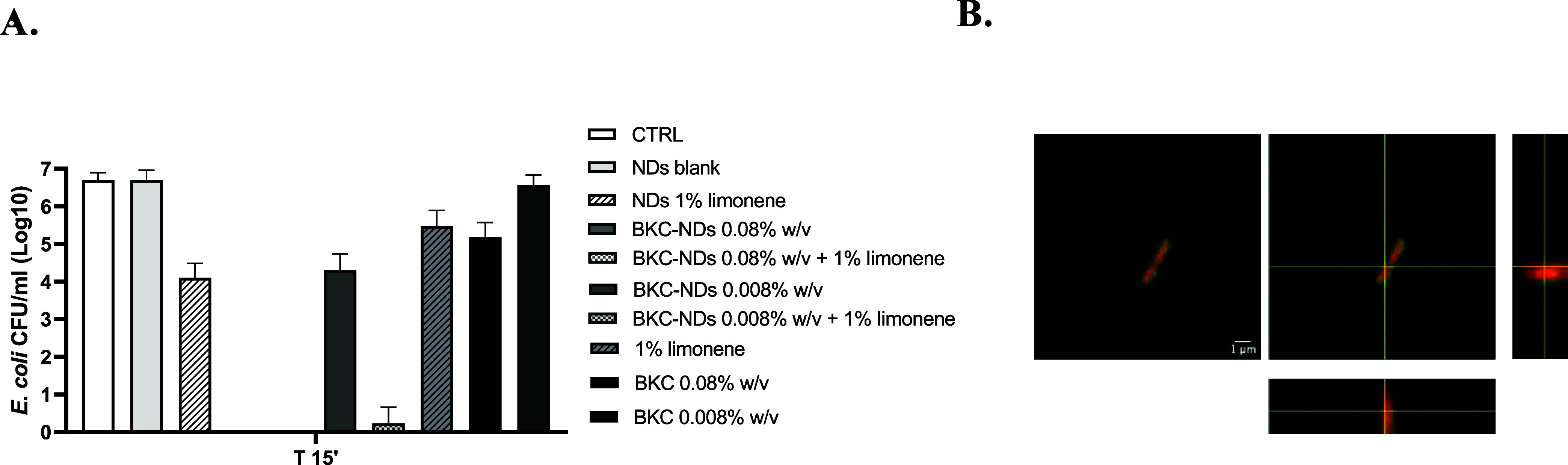
Limonene and the NDs formulation interact with bacterial membrane and enhance BKC antimicrobial effect. (A) Reduction of *E. coli* expressed as Log_10_ of CFU/mL in the presence of BKC-NDs at 0.008 and 0.08% w/v, without or with limonene, NDs 1% limonene, blank NDs, and free limonene (1%) or BKC (0.08 and 0.008% w/v). Data are expressed as mean ± SD of three independent experiments. (B) Confocal image with orthogonal projections of coumarin-blank NDs (green) and *E. coli* stained with propidium iodide (red). The Blank-NDs colocalize (orange) on the surface of the bacteria. Scale bar: 1 μm.

Limonene at low concentration (1%), encapsulated in NDs exhibited antimicrobial activity against *E. coli* but not against *S. aureus* (Table 7). This selective effect can be explained by the enhanced bioavailability and the selective membrane delivery conferred by nanoencapsulation. NDs promote efficient dispersion of limonene and facilitate its interaction with the Gram-negative outer membrane, leading to destabilization of lipopolysaccharides (LPS), increased permeability, and leakage of intracellular contents.^26,45^ Pure limonene shows a MIC of about 20 mL/L (2% v/v) against *S. aureus*,^27^ or an MBC of 25% v/v with free limonene on *S. aureus* compared to *E. coli* (3.12% v/v);^46^ moreover, lemon peel essential oil typically exhibits MBC values of 5% v/v on *S. aureus*.^47^ Sonu et al. found that there was no significant difference in the antimicrobial activity of limonene nanoemulsion at 5% compared to limonene dissolved in DMSO against different tested microorganisms.^48^ In our experimental setting the limonene activity was achieved with concentrations far lower than those typically required in previous studies, highlighting the efficiency of nanoencapsulation in potentiating antimicrobial action. Indeed, it was demonstrated that the Blank-NDs formulation possesses a remarkable *per se* the capacity of interact with the membrane of the bacteria (Figure 7, Panel B), facilitating the contact and the internalization of the active molecules. This result is in agreement with the enhanced antimicrobial effect observed for NDs 1% limonene (a reduction of about 2.5 Log in viable bacteria in NDs compared to a reduction of about 1.5 log with free limonene) and justify the higher efficiency of the BKC-NDs formulations compared to the free BKCs. Further investigations are required to assess whether a fine-tuning of BKC and limonene ratios can result in a synergistic antimicrobial effect also on Gram-positive bacteria (e.g., *S. aureus*). Nevertheless, even in case of lack of additive effect of limonene to the antimicrobial capacity of the formulation, the essential oil maintains its effect as fragrance, as commonly employed in many commercial disinfectants.

Furthermore, limonene suffers from oxidative instability that can promote skin irritation.^31^ It is widely accepted in the literature that the encapsulation of limonene in nanosized formulations protects the essential oil structure from degradation.^32^ Albeit the concentration of limonene in our NDs is what is commonly used in several commercially available formulations as fragrance, further studies are required to confirm whether the proposed NDs prevent limonene oxidation, reducing its allergenic or irritative potential.

To investigate biocompatibility of formulations, cytotoxicity test on human fibroblasts was performed. Both free BKC and BKC-loaded nanodroplets were toxic at 12.5 μg/mL (about 10% for free BKC and 20% for BKC-NDs of cell viability). Free BKC and BKC-NDs at 6.2 μg/mL reduced cell viability of about 50%, while at concentrations ranging from 3.1 to 0.78 μg/mL BKC-NDs maintained a high cell viability (Figure 8). Nanoencapsulation did not substantially mitigate BKC-associated cytotoxicity. BKC exerts cytotoxicity primarily through cationic surfactant-mediated membrane disruption, and encapsulation in an oil-in-water nanodroplet does not sufficiently reduce its bioavailability or membrane affinity to markedly alter its toxicological profile. This finding underscores that, while nanoencapsulation may modulate antimicrobial performance, it does not reduce the known cytotoxicity of cationic surfactants such as BKC or introduce additional risks. For these reasons, the *in vitro* cytotoxicity data should be interpreted as intrinsic to BKC itself and not as a limitation for the safe use of the nanodroplet formulation in environmental contexts.^10^

**Figure 8:**
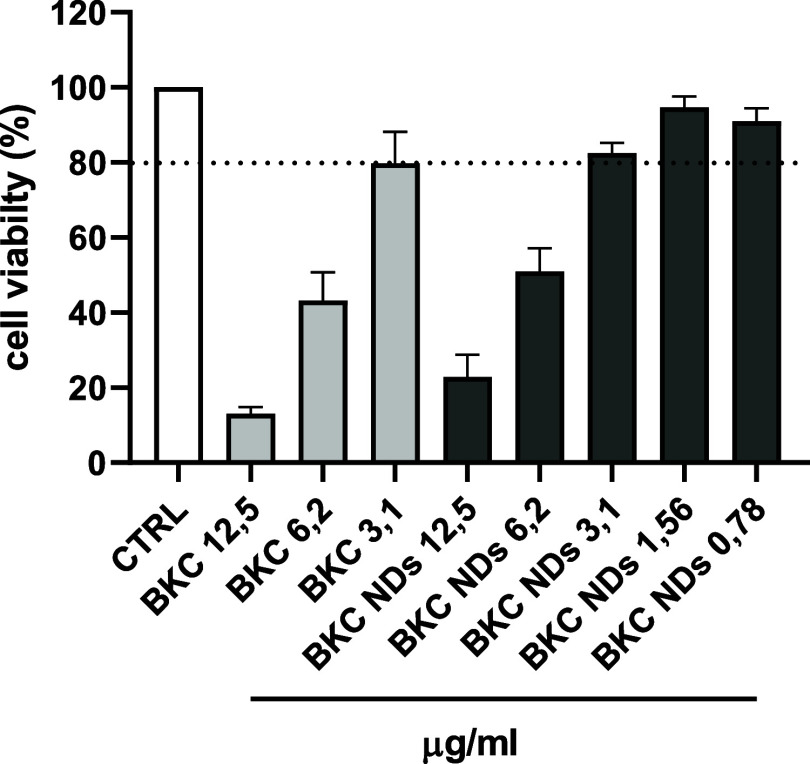
Cytotoxicity on HFFs using MTT assay after exposure to different concentrations of free BKC and BKC-NDs at 24 h. Data are expressed as mean ± SD of three independent experiments and expressed as % of viable cells compared to untreated cells set as 100% cell viability.

## 4 Conclusions

A new spray disinfectant based on BKC in the form of BKC-NDs was developed for household use, with possible application as a medical device. Currently, the industrial production of disinfectants and cleaning agents imposes a considerable environmental and social burden as a result of gas emissions and chemical consumption. In particular, BKC, which remains one of the most widely employed disinfectants, is receiving growing regulatory attention related to its toxicity. Consequently, there is an emerging demand for sustainable, cost-effective, and easy-to-produce spray disinfectant based on QACs. Here, we were able to formulate a solvent-free spray disinfectant based on BKC. The formulation is easy to scale up, as it relies on low-cost ingredients and can be processed using standard industrial equipment, such as high-shear mixers. The novelty of this formulation lies in exploiting the ability of BKC to localize at the oil–water interface, enabling the formation of stable nanoemulsions (BKC-NDs) that enhance the overall antimicrobial performance of the system. In addition, limonene was incorporated as a natural antimicrobial agent to further boost efficacy while providing a pleasant fragrance, ultimately allowing a reduction in the required BKC concentration and addressing concerns related to its toxicity.

Indeed, the optimized spray contains 0.08 g of BKC per 100 mL. In contrast, quaternary ammonium compounds are typically employed in commercial disinfectant formulations at concentrations ranging from approximately 1 up to 2.4 g of QACs per 100 mL. This comparison clearly indicates that the amount of BKC used in the present work is substantially lower than that commonly found in standard QACs-based disinfectants.

This is possible due to the capability of the NDs to interact with the surface of the bacteria, promoting the effective localization of the active ingredients with microorganisms, without raising concerns for cytotoxicity on human cells. Overall, this work presents an effective, eco-friendly spray disinfectant that reduces synthetic chemical use while improving the antimicrobial performance of BKC.

## Supplementary Material



## Notes

Critical micellar concentration of BKC and Tween 20; Quantification of limonene content in the formulations (PDF)

## Abbreviations

BKC: benzalkonium chloride
BSA: bovine serum albumin
CFU: bacterial cell count
CMC: critical micelle concentration
DFP: decafluoropentane
EE: encapsulation efficiency
HFF: human foreskin fibroblasts
LC: loading capacity
LPS: lipopolysaccharides
MIC: minimum inhibitory concentration
MBC: minimum bactericidal concentration
NDs: nanodroplets
QACs: quaternary ammonium compounds
PDI: polydispersity index
RF: reduction factor
γ: surface tension
ζ: ζ potential
