# Supplementary material for: Exploiting Greener Formulations of Benzalkonium Chloride with Enhanced Antibacterial Properties
Source: ACS Omega. 2026 Mar 11;11(11):17354–65. doi: 10.1021/acsomega.5c10270 (PMC13019223; doi:10.1021/acsomega.5c10270)
Supplement: Supplementary file 1 [file ao5c10270_si_001.pdf]

# Supporting Information for

# Exploiting greener formulations of benzalkonium chloride with enhanced antibacterial properties

*Chiara Molinar[1], Giulia Vigna[2], Sara Scutera[2], Tiziana Musso[2], Anna Scomparin[1*], Roberta Cavalli[1]*

1. Department of Drug Science and Technology, University of Turin, Via P. Giuria 9, 10125, Torino

2. Department of Public Health and Pediatric Sciences, University of Turin, Via Santena 9, 10126, Torino

1. **METHODS**.

1.1 Measurement of Critical Micelle Concentration values of surfactants

The Critical Micelle Concentration (CMC) values of Tween 20 and BKC were measured using a Du Noüy ring tensiometer. The surface tension ($\gamma$) of surfactant solutions at increasing concentrations was measured. A graph of surface tension vs. concentration was plotted, and the CMC value was determined as the concentration corresponding to the breakpoint in the slope of the curve. Measurements were performed in triplicate.

1.2 Quantitative determination of Limonene with HPLC

Limonene concentration was quantified using a Shimadzu HPLC system equipped with a UV/Vis detector and a reverse-phase C18 column (150 × 4.6 mm, 5 µm; Agilent 5 TC-C18). The column was maintained at 25 °C, with a 20 µL injection volume. The mobile phase consisted of MeOH/water (85:15, v/v) at a flow rate of 1 mL/min. Detection was performed at 204 nm. Calibration was linear in the range 0.5–100 µg/mL. Prior to injection, samples were filtered through a 0.22 µm membrane.

2. **RESULTS**.

2.1 Measurement of Critical Micelle Concentration values of surfactants

The plot of surface tension vs. concentration showed a decrease in surface tension as the surfactant concentration increased. The CMC values of BKC and Tween 20 were determined at the concentration in the point of intersection of the two equations, where the slope of the line undergoes a significant change. Tween 20 showed a CMC value of 0.01 mg/mL and BKC of 1.19 mg/mL (Figure S1).

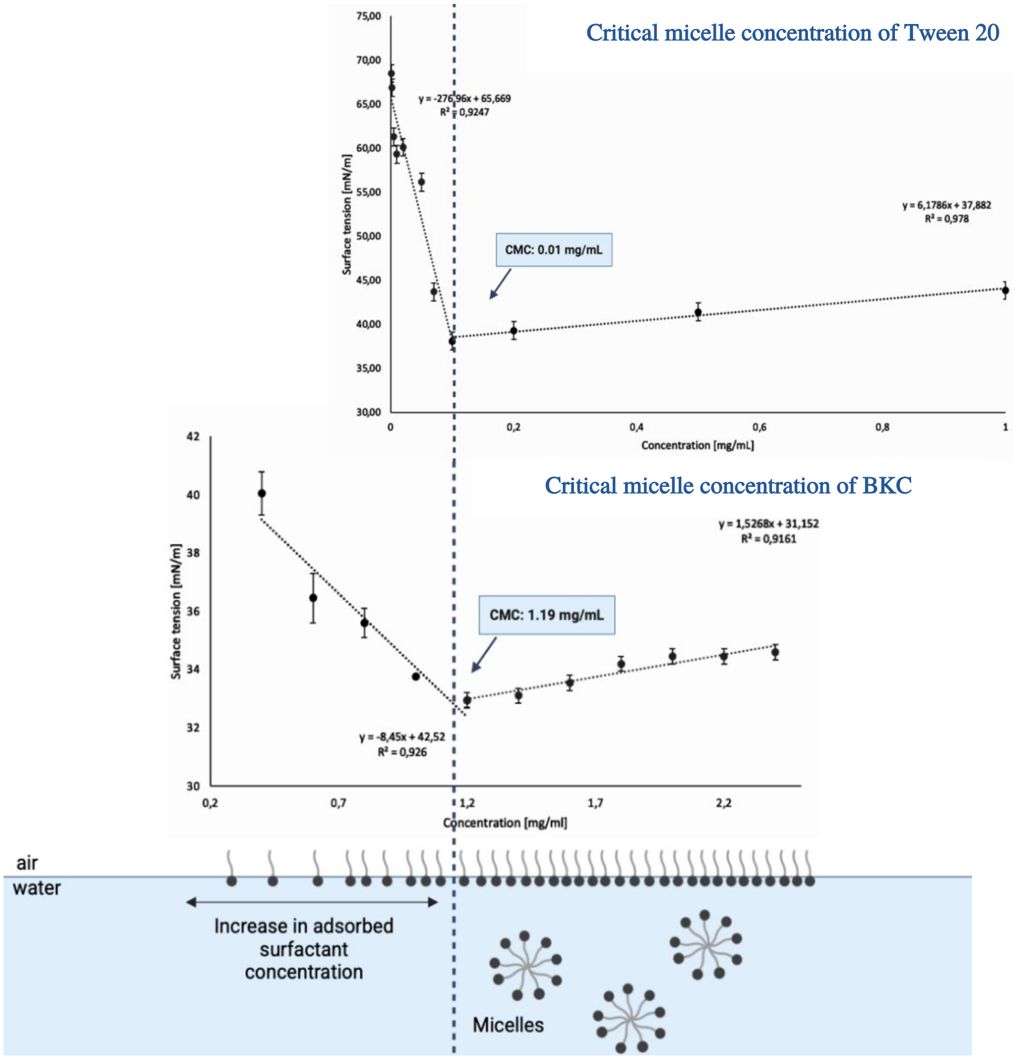


**Figure S1:** Critical micelle concentration values of BKC and Tween 20

2.2 Limonene was quantified via HPLC

The limonene peak was detected at 7.60 min (Figure S2). The calibration curve was linear in the range 0.5–100 µg/mL, with a regression coefficient of 0.999. Limonene was successfully encapsulated in NDs with a high EE% values as reported in Table S1.

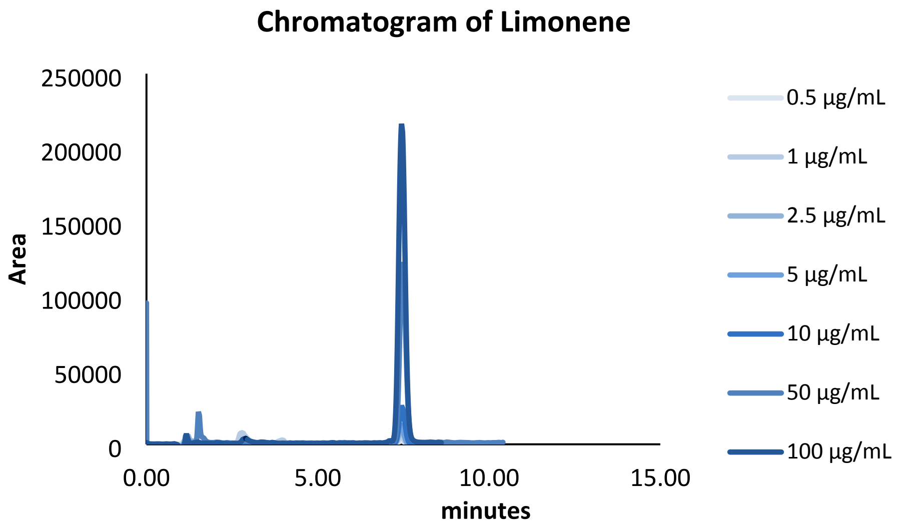


**Figure S2:** Limonene chromatogram

**Table S1:** EE% and LC% values of limonene in BKC-NDs with and without DFP at 0.008%, 0.08% and 2.4% w/v

| Formulations | Encapsulation efficiency (EE%) ± SD | Loading capacity (LC%) ± SD | Limonene concentration (mg/mL) ± SD |
|---|---|---|---|
| BKC-NDs 0.008% w/v | 102.00 ± 1.01 | 0.86 ± 0.01 | 8.59 ± 0.04 |
| BKC-NDs 0.08% w/v | 104.00 ± 1.20 | 1.13 ± 0.01 | 11.34 ± 0.11 |
| BKC-NDs 2.4 % w/v | 90.40 ± 1.88 | 0.76 ± 0.02 | 7.60 ± 0.15 |
| BKC-NDs 0.008% w/v (w/o DFP) | 103.00 ± 0.27 | 0.87 ± 0.002 | 8.73 ± 0.02 |
| BKC-NDs 0.08% w/v (w/o DFP) | 106.00 ± 1.17 | 0.89 ± 0.01 | 8.91 ± 0.10 |
| BKC-NDs 2.4 % w/v (w/o DFP) | 105.00 ± 1.27 | 0.90 ± 0.01 | 8.80 ± 0.11 |

2.3 Contact angle images of BKC-NDs

BKC-NDs demonstrated improved surface wettability, showing lower contact angles than aqueous BKC solutions, as reported in Figure S3.

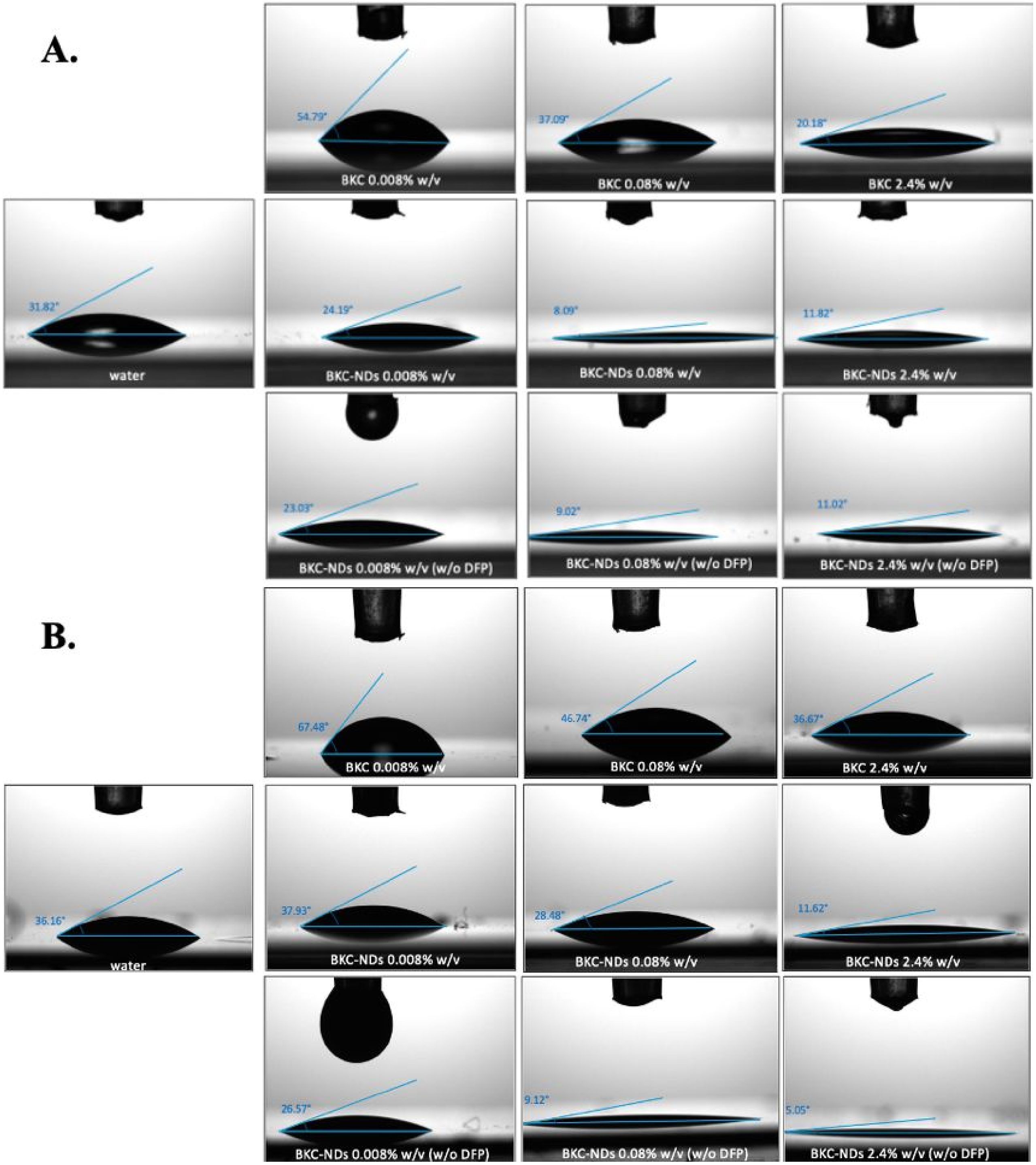


Figure S3: Contact angle images of BKC-NDs on different surfaces mimicking household cleaning scenarios: (A) glass surface and (B) plastic surface

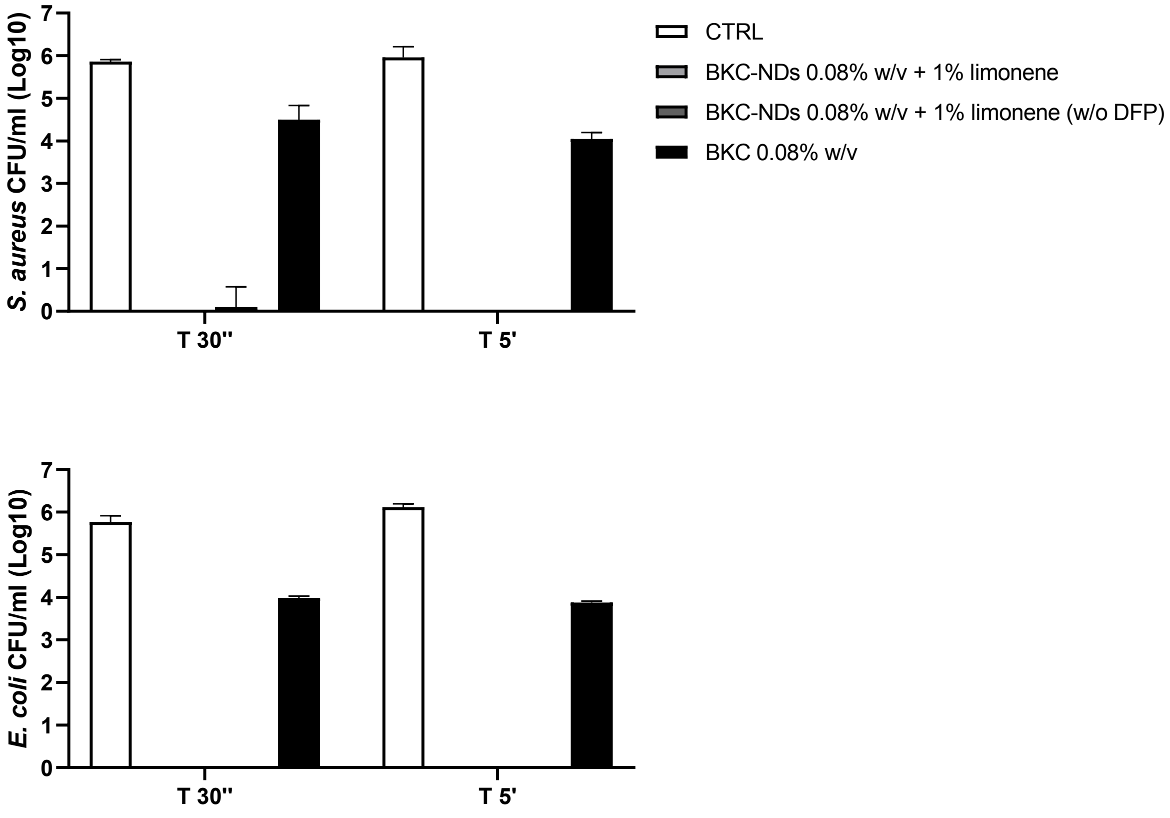


**Figure S4:** Reduction of *S. aureus* (upper panel) and *E. coli* (lower panel) expressed as $Log_{10}$ of CFU/mL in the presence of BKC-NDs at 0.08% w/v, 30 s and 5 min contact-time at 30°C. Data are expressed as mean ± SD of three independent experiments

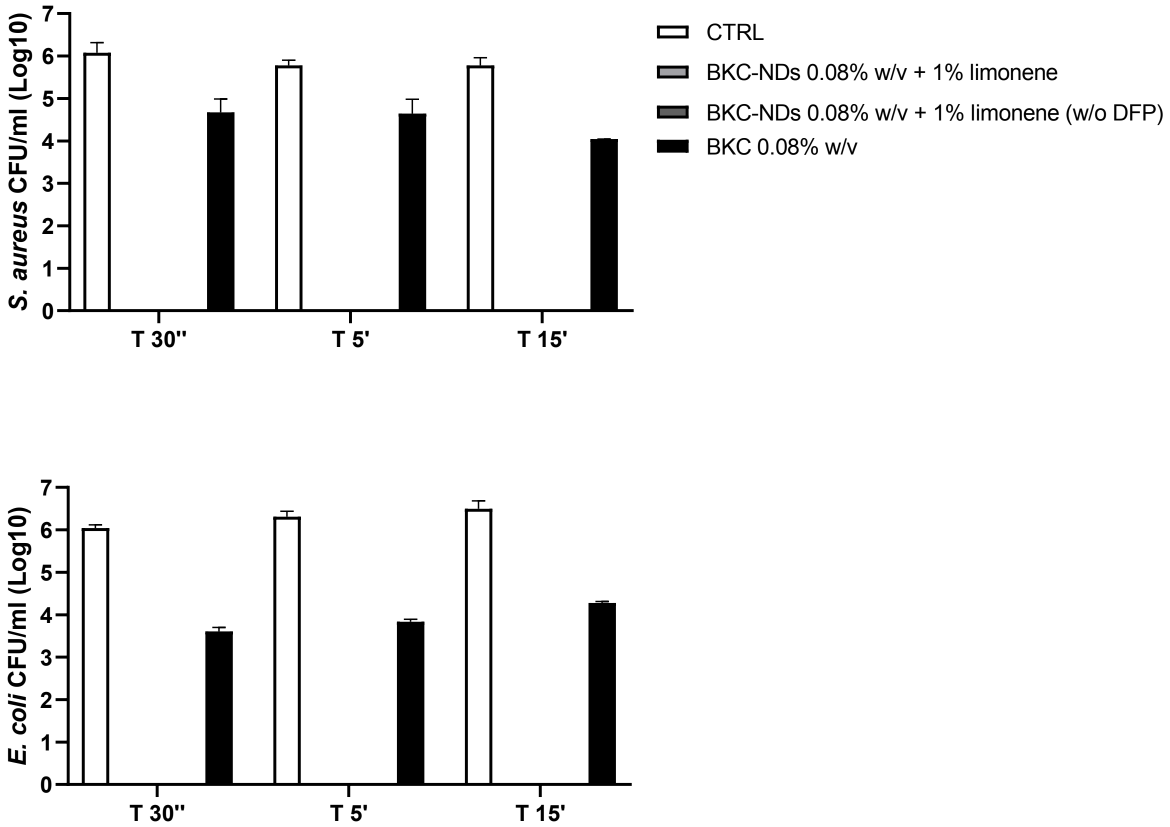


**Figure S5:** Reduction of *S. aureus* (upper panel) and *E. coli* (lower panel) expressed as $Log_{10}$ of CFU/mL in the presence of BKC-NDs at 0.08% w/v, 30 s, 5 min and 15 min contact-time at 20°C. Data are expressed as mean ± SD of three independent experiments.
